# Vigorous Exercise in Patients with Hypertrophic Cardiomyopathy: Results of the Prospective, Observational, Multinational, “Lifestyle and Exercise in HCM” (LIVE-HCM) Study

**DOI:** 10.1001/jamacardio.2023.1042

**Published:** 2023-05-17

**Authors:** Rachel Lampert, Michael J. Ackerman, Bradley S. Marino, Matthew Burg, Barbara Ainsworth, Lisa Salberg, Maria Teresa Tome Esteban, Carolyn Y. Ho, Roselle Abraham, Seshadri Balaji, Cheryl Barth, Charles I. Berul, Martijn Bos, David Cannom, Lubna Choudhury, Maryann Concannon, Robert Cooper, Richard J. Czosek, Anne M. Dubin, James Dziura, Benjamin Eidem, Michael S. Emery, NA Mark Estes, Susan P. Etheridge, Jeffrey B. Geske, Belinda Gray, Kevin Hall, Kimberly G. Harmon, Cynthia A. James, Ashwin K. Lal, Ian H. Law, Fangyong Li, Mark S. Link, William J. McKenna, Silvana Molossi, Brian Olshansky, Steven R. Ommen, Elizabeth V. Saarel, Sara Saberi, Laura Simone, Gordon Tomaselli, James S. Ware, Douglas P. Zipes, Sharlene M. Day

**Affiliations:** a Yale School of Medicine, New Haven, CT, USA; b Mayo Clinic; c Cleveland Clinic Heart, Vascular and Thoracic Institute; d Lurie Children’s Hospital; e Arizona State University; f Hypertrophic Cardiomyopathy Association; g St George’s Hospital NHS Foundation Trust/St George’s University of London; h Brigham and Women’s Hospital; i Johns Hopkins University; j Oregon Health and Science University; k Childrens National Hospital; l PIH good Samaritan; m Northwestern University Feinberg School of Medicine; n University of Michigan; o Liverpool Heart and Chest Hospital/Liverpool John Moores University; p Cincinatti Childrens’ Hospital; q Stanford School of Medicine; r Indiana University School of Medicine; s Tufts Medical Center; t University of Pittsburgh Medical Center; u Primary Children’s Hospital; v Royal Prince Alfred Hospital/Faculty of Medicine and Health, University of Sydney, Australia; w University of Washington, Johns Hopkins University; x University of Iowa; y University of Texas, Southwestern; z University College, London; aa Baylor College of Medicine, Texas Children’s Hospital, Houston, TX, USA; ab St Luke’s Health System; ac Albert Einstein College of Medicine; ad I National Heart and Lung Institute & MRC London Institute of Medical Sciences, Imperial College London/Royal Brompton & Harefield Hospitals, Guy’s and St. Thomas’ NHS Foundation Trust

## Abstract

**Importance:**

Whether vigorous intensity exercise increases risk of ventricular arrhythmias in individuals with hypertrophic cardiomyopathy (HCM) is unknown.

**Objective:**

To determine whether engagement in vigorous exercise is associated with increased risk for ventricular arrhythmias and/or mortality in individuals with HCM. The a priori hypothesis was that participants engaging in vigorous activity were not more likely to have an arrhythmic event or die than those who reported non-vigorous activity.

**Design:**

Investigator-initiated, prospective observational study. Participants were enrolled 5/2015 to 2/2019, median follow up of 38 months completed 2/2022. Participants were categorized according to self-reported levels of physical activity: sedentary, moderate, or vigorous intensity exercise.

**Setting:**

Multicenter, observational registry with recruitment at 42 high-volume HCM centers in the United States and internationally. Patients could also self-enroll through the central site.

**Participants:**

Individuals 8-60 years old diagnosed with HCM, or genotype-positive without left ventricular hypertrophy (phenotype-negative), without conditions precluding exercise, were enrolled.

**Exposure:**

Amount and intensity of physical activity

**Main Outcomes and Measures:**

The primary pre-specified composite endpoint included death, resuscitated sudden cardiac arrest, arrhythmic syncope, and appropriate ICD shocks. All outcome events were adjudicated by an Events Committee blinded to the patient’s exercise category.

**Results:**

Among the 1660 participants, 252 were classified as sedentary (15%), 709 (43%) participated in moderate, and 699 (42%) in vigorous intensity exercise (259 competitively, 37% of vigorous group). Seventy-seven individuals (4.6%) reached the composite endpoint: 44 (4.6%) of those classified non-vigorous, and 33 (4.7%) classified vigorous, with corresponding rates of 15.3 and 15.9 per 1000 person-years. In multivariate Cox regression analysis of the primary composite endpoint, individuals engaging in vigorous exercise did not experience a higher rate of events compared to the non-vigorous group with adjusted hazard ratio (HR) of 1.01. The upper 95% one-sided confidence level (UCL) is 1.48, below the pre-specified boundary of 1.5 for non-inferiority.

**Conclusions and Relevance:**

Among individuals with HCM, or those who are genotype-positive/phenotype -negative, who are managed in experienced centers, those exercising vigorously did not experience a higher rate of death or life-threatening arrhythmias than those exercising moderately or the sedentary. These data will inform discussion between the patient and their expert care provider around exercise participation.

## Introduction

Exercise has well-established physical and mental health benefits and is an integral part of life for millions of people worldwide. However, for individuals with hypertrophic cardiomyopathy (HCM), the possibility that physical activity may heighten risk of sudden cardiac death (SCD) has led to exercise restriction and disqualification from competitive sports. HCM is defined by unexplained left ventricular hypertrophy (LVH) and has a worldwide prevalence of ~1:500 individuals.^1^ It is the most common genetic cardiomyopathy and is inherited as a Mendelian trait in ~50% of patients, predominantly due to pathogenic variants in genes that encode sarcomeric proteins. In others it is expressed as a complex trait with contributions from both polygenic and acquired factors. Some patients with HCM may experience symptoms of heart failure, atrial and/or ventricular arrhythmias, while others have normal longevity and good quality of life.^2^ As HCM is a well-recognized cause of SCD in previously-undiagnosed young individuals, including athletes,^3^ there has been intense debate over the last four decades over medical recommendations around participation at any level of physical activity, including recreational exercise and competitive sports, for individuals diagnosed with HCM. U.S. and European consensus guidelines dating back to 19854 have recommended against vigorous recreational physical activity and ineligibility for sports participation for all patients with HCM – a conservative approach in light of the lack of any outcomes data. However, restriction from exercise has adverse consequences. Individuals with HCM exercise less than the general population, and report a higher prevalence of obesity, heightened anxiety, and reduced emotional well-being.^5, 6^

Recommendations are evolving, with the most recent AHA/ACC 2020 guidelines^7^ now recognizing the benefits of mild-moderate intensity recreational exercise in patients with HCM. This new Class I recommendation was supported by evidence from the RESET-HCM clinical trial, in which adult patients who followed prescriptions of moderate intensity exercise showed significant improvements in exercise capacity and physical functioning.^8^ Although the study was underpowered for safety, there were no major adverse events and no increase in non-fatal arrhythmias in the exercise trained group compared to the usual activity group. The 2020 ACC/AHA HCM guidelines, ^7^ and the 2019 European guideline^9^, also introduced a new recommendation that participation in vigorous recreational exercise and competitive sports participation could be considered using a framework of shared decision-making^10^ for all^7^ or some^9^ patients with HCM. However, the ACC/AHA recommendation remains a “Class 2B” due to limited data on whether vigorous physical activity increases risk of ventricular arrhythmic events in patients with HCM. There remains uncertainty even for those who carry a genetic variant without left ventricular hypertrophy (LVH) (i.e. genotype positive/phenotype negative), particularly in light of post-mortem studies that have identified pathogenic genetic variants in 5-10% of sudden cardiac death victims without structural abnormalities at autopsy^11, 12^ The guidelines prioritize thess knowledge gaps as a major unmet need. The prospective, multinational, NIH-funded LIVE-HCM study was designed to provide data to inform patient-provider decisions, with its primary objective to determine whether engagement in vigorous exercise, including competitive sports, is associated with increased risk for life-threatening ventricular arrhythmias and/or mortality in individuals with HCM.

## Methods

### Study Design

This was an investigator-initiated prospective observational study. This study was approved by the Yale Human Investigation Committee and by institutional review boards of participating sites. All patients provided signed informed consent.

### Patients and recruitment

Individuals 8-60 years old with a diagnosis of overt HCM (phenotype positive), or genotype-positive/phenotype-negative, were eligible to participate. Individuals were excluded if they had conditions precluding vigorous exercise (advanced HCM-related symptoms (New York Heart Association class III or IV), or non-HCM-related conditions), as were those with LVH due to syndromic conditions or infiltrative disease. Individuals unable to complete online or phone questionnaires due to language or cognitive barriers were also excluded.

Patients were enrolled either through participating high-volume HCM centers (N=42), in the United States, United Kingdom, Canada, Australia, and New Zealand, or through contacting the central site (Yale) directly (“self-enrolled”, 24%), between 5/2015 and 2/2019. Information was disseminated to patients by the advocacy organization Hypertrophic Cardiomyopathy Association (HCMA,) other patient group Internet sites and mailing lists, and via mailings to physicians. For site-enrolled patients, diagnosis of HCM and eligibility were confirmed by the site. For self-enrolled patients, after consent and medical release forms were signed, diagnosis and eligibility were confirmed by chart review, interpretation of echocardiogram images by a Core Lab at the Mayo Clinic or of cardiac magnetic resonance (CMR) images at the University of Michigan. Derivation of the final cohort of 1660 participants is shown in Supplemental Figure 1.

### Study procedures

Patients who consented were contacted by the central study team and sent a link to online questionnaires via a REDcap database. Records were obtained from sites and via medical release forms from physicians. Demographic, clinical, and genetic data were abstracted and entered into the REDcap database. Participants received a link to a brief survey querying the occurrence of outcome events every six months. If outcome events were reported, study coordinators contacted participants for details and records were obtained. If patients did not complete the planned 36 months of outcome surveys, records were obtained from sites or physicians to assess for events. Vital status was confirmed for all patients via sites or national death registries. Follow up was complete in 2/2022.

### Baseline Assessments

The primary independent variable, exercise level, was based on the Minnesota Leisure Time Activity Questionnaire,^13^ which has been validated against both direct^14, 15^ (treadmill exercise performance, accelerometry) and indirect, (frequent detailed activity records) ^15^ criteria, and has high test-retest reliability^15, 16^. Participants identified physical activities performed in the past year, indicating the months per year, times per month, and the average time per event they performed each activity. Activities were assigned a metabolic equivalent (MET) intensity level defined as the oxygen cost of physical activity in ml·kg^-1^·min^-1^ divided by the oxygen cost at rest (3.5 ml·kg^-1^·min^-1^). MET values were obtained from the 2011 Compendium of Physical Activities.^17^ Participation in at least one activity at METS > 6.0 for > 60 hours per year, was categorized as vigorous. ^18^ This level of intensity is beyond what has been recommended for patients with HCM for exercise. ^19^ Those participating in activities at METS > 4 and < 6 for > 60 hours per year, but not meeting criteria for vigorous, were categorized as moderate, and those not meeting either of these criteria, as sedentary. Those in moderate and sedentary groups were categorized as non-vigorous for the primary analysis. Patients were also queried about current participation in competitive level athletics. A subset of those meeting criteria for vigorous exercise performed at least one activity competitively, and were categorized as the vigorous-competitive subgroup.

### Outcomes

All outcome events were reviewed and adjudicated by a Clinical Events Committee blinded to the patient’s exercise category. The primary pre-specified composite endpoint included death, resuscitated sudden cardiac arrest (SCA), syncope adjudicated to be definitely or likely arrhythmic, and appropriate ICD shocks, with or without syncope. All ICD shock events were reviewed by two electrophysiologists. If electrograms could not be obtained, reports of electrograms from the record were reviewed. Deaths were classified as sudden-arrhythmic/cardiac (SCD), non-sudden cardiac, or non-cardiac using standard definitions.^20^

### Statistical analysis

#### Sample Size and Power

Sample size was determined using the Log Rank Non-Inferiority module of PASS (Kaysville, UT). Original sample size was estimated at 2250 participants based on a 21 month recruitment period, total study time of 57 months, an event rate of 11.5% over 3 years, ^21, 22^ 10% dropout and 90% power at the 0.05 one-sided significance level to declare non-inferiority of vigorous and moderate to sedentary at the upper boundary for a hazard ratio (HR) of 1.5. These estimates were based on expected proportions of exercise intensity based on published data in patients with HCM and the general population: 25% vigorous, 25% moderate and 50% sedentary. ^5^, ^23^ However, the ratio of individuals performing vigorous compared to sedentary and moderate activity was higher than expected. Therefore, prior to analysis, we revised the comparison to compare the vigorous group to the moderate and sedentary groups combined, defined for this analysis as “non-vigorous”. Our achieved sample size (699 vigorous, 961 non-vigorous) provides 91% power at the one-sided 0.05 significance level to declare non-inferiority of vigorous to non-vigorous at the 1.5 HR boundary.

#### Selection of the non-inferiority boundary

For studies comparing interventions, it is customary to use data comparing the “standard” intervention to placebo, but analogous data are not available for exercise risk. Our rationale for using a HR of 1.5 as the upper bound was two-fold. First, consensus, derived from clinical judgment amongst our steering committee and investigators, was that 1.5 represented a clinically relevant margin. Second, 1.5 has been suggested as appropriate in trials in which no data are available on standard-placebo differences.^24^ This margin is within the range reported in large trials in the literature.

#### Analysis of Primary Outcome

The primary outcome variable is a composite endpoint of time to first of either death, SCA, appropriate ICD shock (for ventricular arrhythmia) or arrhythmic syncope. Time was determined from date of enrollment to date of either first event, transplant (censor; n= 4 sedentary, 7 moderate, 3 vigorous)) or of date last survey or clinical follow up (censor). Kaplan-Meier plots were constructed showing event-free survival. Our non-inferiority hypothesis was that participants reporting vigorous activity at enrollment were no more likely to have an event than those reporting non-vigorous activity (i.e. not inferior within the specified margin based on a HR of 1.5). Event likelihood was compared between groups using Cox regression including activity level (vigorous vs non-vigorous activity) as well as covariates for age, sex, race, recruitment method (site or self), age at diagnosis and presence of an ICD. Linear contrasts were estimated to compare the hazards of the vigorous group with the moderate/sedentary group. A non-inferiority boundary for the hazard ratio of 1.5 was used with non-inferiority concluded when the upper boundary of the 95% one-sided Wald confidence interval was less than 1.5. Pairwise non-inferiority comparisons of vigorous to moderate and sedentary groups are also provided per the original analysis plan. Post hoc sensitivity analyses were performed excluding first, genotype-positive/phenotype-negative individuals, and then, participants with exertional dyspnea or chest pain (NYHA II). A similar analysis was performed to compare the vigorous-competitive subgroup to non-vigorous group. Subsequent models included controlling for known clinical risk factors for sudden cardiac death^7^ which differed between groups (effect size > 12%) including history of arrest in the entire overt HCM group, followed by, septal thickness, (excluding those with apical variant.) To determine, among those with overt HCM, whether apical variant HCM, or presence of a pathogenic mutation, impacted any associations of exercise with the composite endpoint, interaction testing was performed.

## Results

### Patient population

Demographic, clinical, and genotype data are shown for vigorous and non-vigorous groups (Table 1) and with the non-vigorous divided into moderate and sedentary groups (Supplemental Table 1). Among the 1660 participants, 60% male, mean age 39 +/- 15 years, 252 were categorized as sedentary (15%), 709 (43%) participated in moderate intensity exercise, and 699 (42%) participated in vigorous intensity exercise. Among those engaging in vigorous exercise, 259/699 (37%) participated competitively and 440 (63%) non-competitively. Overall, 60% were male. Ninety two percent had overt HCM (ie, phenotype positive), among whom 24% reported exertional dyspnea (New York Heart Association (NYHA) Class II). Those engaging in vigorous exercise were on average younger, more likely to be male, more likely genotype-positive/phenotype-negative, and less likely to be NYHA II. There was no significant difference in the rate of prior ICD implants. The prevalence of risk factors for sudden cardiac death (SCD) - - history of syncope or non-sustained ventricular tachycardia, family history of SCD, degree of hypertrophy, and presence of late gadolinium enhancement on CMR-- did not differ between groups, nor did presence of an HCM-related pathogenic variant. Median follow-up was 38 months which did not differ between groups. Among the phenotype positive patients, 39% reported that a physician whom they had seen recommended no or light activity only, and in an additional 43%, that competition was recommended to be restricted (total 82%), with analogous recommendations reported by the phenotype negative patients in 19% and 26% respectively (total 45%).

The subgroup of vigorous competitive athletes were younger, more likely to be genotype-positive/phenotype-negative, more likely to have apical HCM, and less likely to have an ICD. Traditional risk factors for SCD did not differ. Forty-seven were age 8-13 participating in leagues, 74 were age 14-22, among whom 54 participated on varsity, junior varsity, or traveling teams, and 138 were age 23-60, participating in leagues or organized events. The most common sports were baseball/softball, running/track, soccer, and basketball (Supplemental Table 2).

### Outcomes

**Figure 1:**
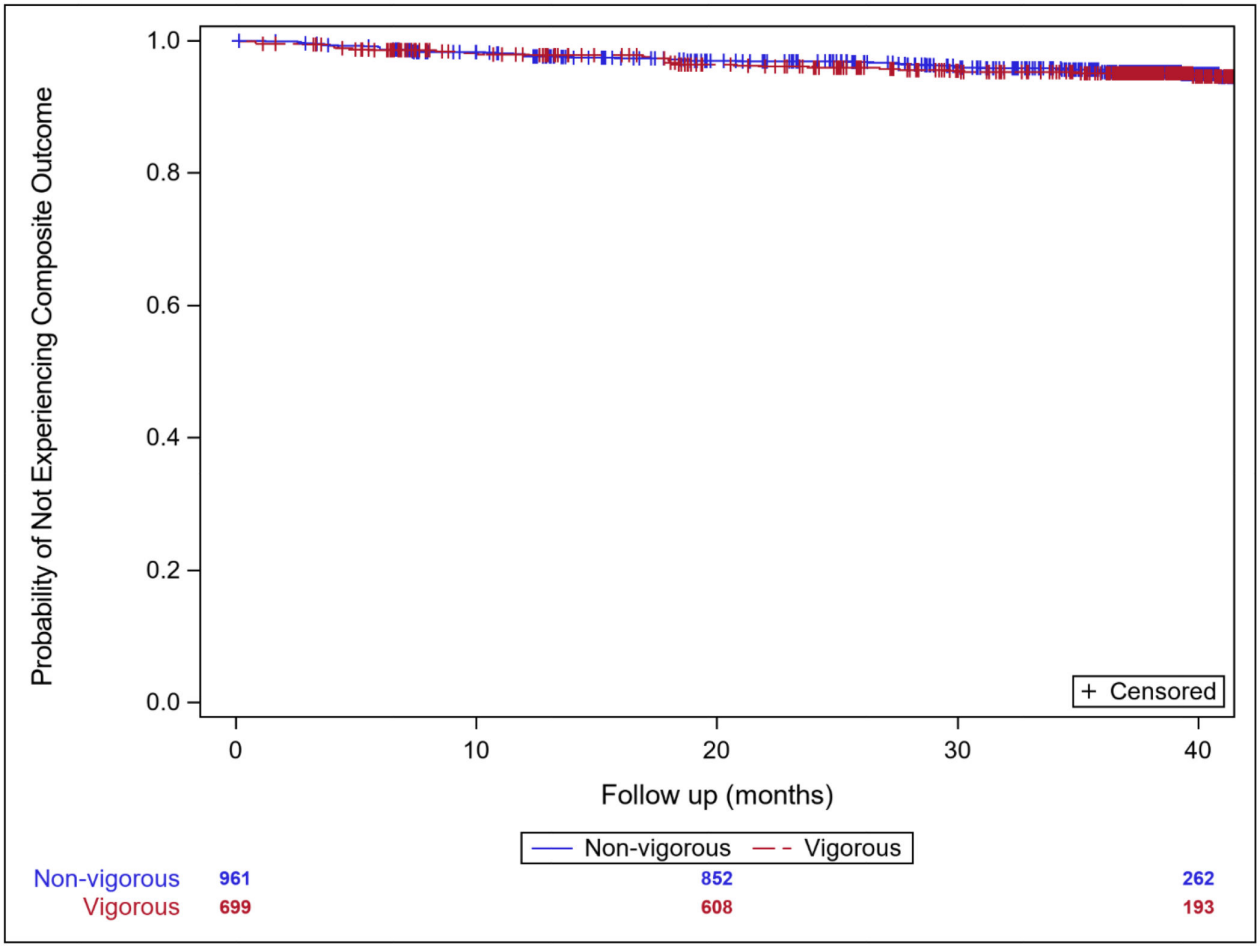
Kaplan-Meier survival curve for freedom from composite endpoint (death, cardiac arrest, appropriate ICD shock, or arrhythmic syncope) by exercise group Vigorous and non-vigorous groups did not differ in freedom from composite endpoint

As shown in Table 2, 77 individuals (4.6%) reached the composite endpoint of death, SCA, appropriate ICD therapy (with or without syncope), or arrhythmic syncope. Forty-four (4.6%) of those classified as non-vigorous, and 33 (4.7%) as vigorous experienced the composite endpoint, with corresponding rates of 15.3 and 15.9 per 1000 person-years. Survival free of events is shown in Figure 1 and Supplemental Figure 2.

Characteristics of participants experiencing SCD (N = 8) or SCA (N = 6) are shown in Table 3. There were no events in individuals without overt HCM. Among the 7 individuals engaged in vigorous exercise who experienced SCA or SCD, 3 SCAs occurred during exercise (2 recreational and 1 competitive) and 2 SCDs and 1 SCA during activities of daily living. Activity at the time of one SCD was unknown. ICDs failed to convert a ventricular arrhythmia in one child in the sedentary group who arrested while standing in line at school, and failed to prevent SCD in one adult in the moderate group, while hunting, and one adult in the vigorous group, while driving. Among the remaining 11 with SCA or SCD, without an ICD, 4 had at least one risk factor for SCD.

### Primary Analysis

**Figure 2:**
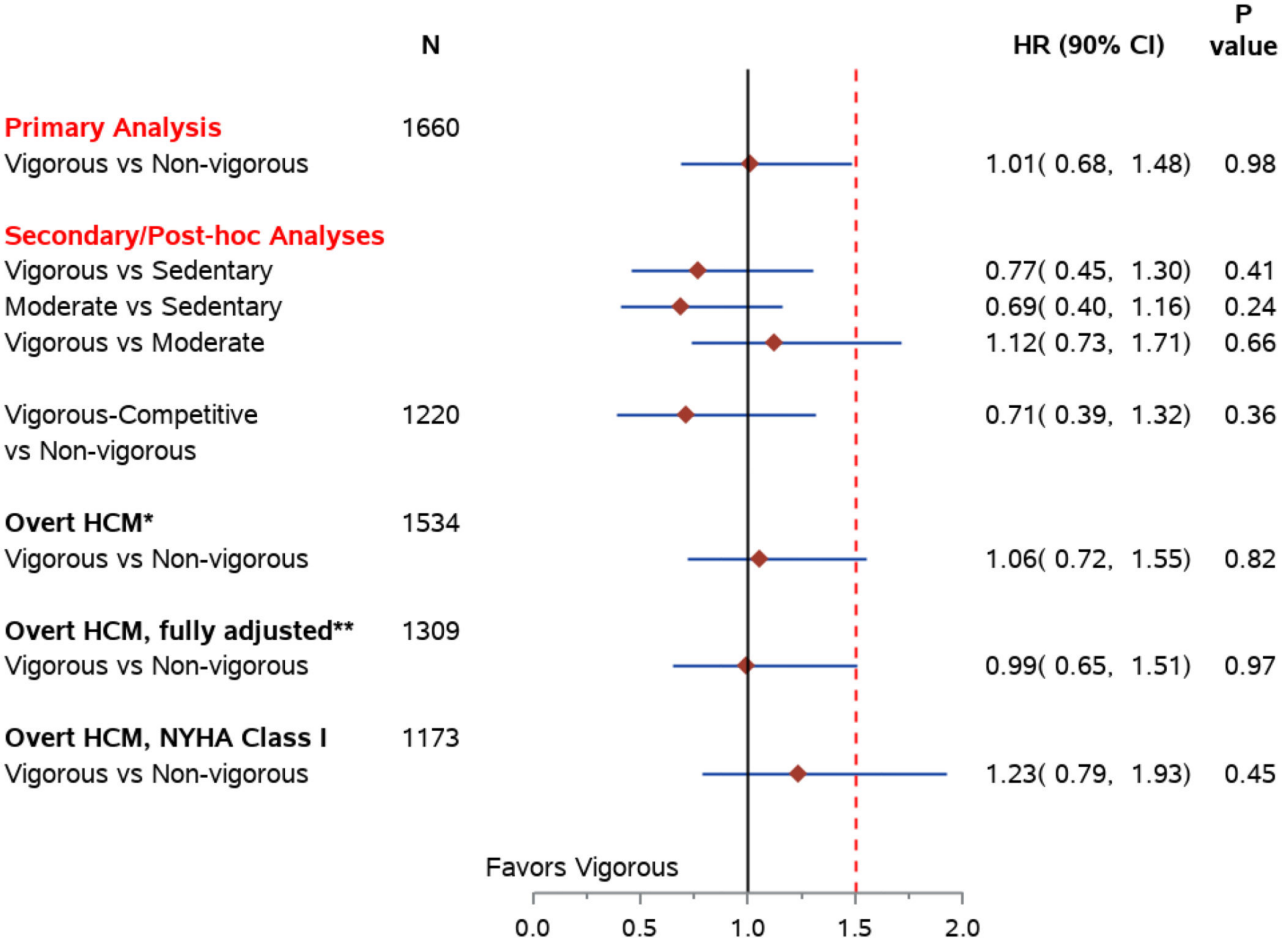
Forest plot for HR (one-sided 95% CI) comparing composite outcomes between exercise groups Hazard ratios for primary, secondary, and post-hoc analyses comparing the composite outcome (death, cardiac arrest, appropriate ICD shock, arrhythmic syncope) between those exercising vigorously and those exercising non-vigorously. 90% two-sided confidence intervals are presented. The upper limits of these intervals correspond to a onesided 0.05 significance level used to evaluate non-inferiority. Primary analysis is shown followed by two secondary analyses: pairwise comparisons of the three groups, and after excluding non-competitive vigorous individuals to compare vigorous-competitive vs non-vigorous. Post hoc analyses are shown of subgroups: First, those with overt HCM (ie, phenotype-positive only), * controlling for pre-specified covariates age, sex, race, recruitment method (site or self), age at diagnosis and presence of an ICD, and ** adding SCD risk factors which differed by an effect size of at least 12% between the groups (history of sudden cardiac arrest and septal thickness) to the model, and finally, after excluding those with exercise-related symptoms (ie, asymptomatic, phenotype-positive only).

In multivariate Cox regression analysis of the primary pre-specified composite endpoint, individuals engaging in vigorous exercise did not experience a higher rate of events compared to those in the non-vigorous group with an adjusted hazard ratio (HR) of 1.01. The upper 95% one-sided confidence level (UCL) is 1.48, below the pre-specified boundary of 1.5 for non-inferiority (Figure 2).

### Secondary and Post-hoc analyses

In a pre-specified secondary analysis, those engaging in vigorous-competitive exercise also did not experience an increased arrhythmic risk compared to the non-vigorous group with HR 0.71, UCL 1.32. (Figure 2). In pre-specified pairwise sub-analyses, moderate exercise was non-inferior to being sedentary. No subgroup was found superior to another.

Post-hoc analyses were performed in subgroups with characteristics which differed by an effect size of >12% between the vigorous and non-vigorous groups. First, genotype-positive/phenotype negative patients were excluded. Among this subgroup of phenotype-positive patients, similar point-estimates for the HR for vigorous activity compared to the non-vigorous group were seen. Controlling for known risk factors for sudden cardiac death which differed between groups (history of arrest, and septal thickness), did not impact the findings. Next, those with NYHA II were excluded. In this subgroup of asymptomatic, phenotype positive patients, findings were similar. In neither the primary nor any of the sub-analyses, was either non-vigorous or vigorous exercise demonstrated to be superior (Figure 2). Among those with overt HCM, there was no interaction between either apical phenotype, or presence of a pathogenic variant, and exercise in association with the composite endpoint.

Although all SCD and SCA events occurred in males (Table 3), the overall occurrence of outcome events did not differ between males (4.57%) and females, (4.79%), nor was there a sex difference in outcomes within the exercise categories.

There were 203 individuals age 14-22 years, among whom 56 individuals were competing in varsity sports/traveling teams, (42 with overt HCM), 50 engaging in other vigorous exercise, either recreationally or at lower levels of competition, and 97 engaging in moderate exercise or who were sedentary. Demographic and clinical characteristics are shown in Supplemental Table 4. There was one outcome event in the varsity/traveling team group, (resuscitated cardiac arrest, see Table 3 and Supplemental Table 5, none in the other vigorous group, and 6 outcome events in the moderate/sedentary group (2 deaths, see Table 3, 3 appropriate ICD shocks, 1 arrhythmic syncope, see Supplemental Table 5,) corresponding to event rates per 1000 person-years of 5.7 (0.8-40.8), 0, and 20.7 (9-46.2) for varsity/traveling, other-vigorous, and moderate-sedentary groups respectively.

## Discussion

In this prospective study of 1660 individuals with HCM or who are genotype-positive/phenotype-negative (8%), those engaged in vigorous exercise did not experience a heightened risk of death, cardiac arrest, appropriate ICD shocks or arrhythmic syncope compared to individuals engaging in low to moderate intensity physical activity. Individuals who were participating in high-intensity competitive sports were also not at heightened risk. Overall absolute event rates were low, with fewer than 5% reaching the composite outcome over 3 years of follow-up. Post hoc analysis limited to those with overt HCM showed a similar HR although non-inferiority was not demonstrated based on the pre-specified boundary. In neither the primary patient population, nor any subgroup comparison, was either vigorous or non-vigorous exercise shown to be safer (superior). Most life-threatening events occurred during activities of daily living. Among the subgroup of 42 younger, highly competitive athletes with overt HCM, there was one event (non-fatal), which did not occur during competitive exercise. In total, these findings do not support universal restriction of vigorous intensity exercise in patients with HCM.

These prospective data challenge long-held beliefs that vigorous and competitive exercise increase the likelihood of arrhythmia for individuals with HCM^4, 25^, which has driven guideline recommendations for 4 decades. These conservative recommendations were based on early observational series showing that HCM was a common cause of SCD among athletes and that most events occurred during exercise^3^. Subsequent series of SCD in athletes have suggested that HCM is accountable for a smaller proportion of events, and that few SCD occur during exercise^26–28^. Recent series of athletes with HCM who continue to participate in sports have shown no adverse events, ^29–31^ with the exception of one report including 2 athletes with HCM who died during high-intensity exercise after returning to play.^32^ In a prior study of athletes with ICDs, including 77 with HCM, the athletes were equally likely to experience ventricular arrhythmias during non-sports-related activity, as during sports. ^33, 34^ A retrospective cross-sectional study of individuals with HCM ^35^ did not show increased ventricular arrhythmias in those with higher cumulative hours of exercise. These studies have been limited however, by small size, older populations, retrospective nature, and/or lack of a control group of less active individuals.

The safety of highly competitive athletics, such as varsity high-school and college competition, for individuals with HCM has been debated. ^36^ In this study, the 42 high school and varsity college athletes with overt HCM had a lower rate of events than moderate/sedentary individuals of similar age. This age-group is too small for meaningful statistical analysis with or without controlling for potential confounding differences. However, it is reassuring that the overall event rate is low, and that the single event was non-fatal and did not occur during competition. Despite these limitations, these data will inform shared-decision making as endorsed by recent guidelines^7^. Ongoing and future studies of highly competitive athletes will also be informative in guiding these decisions.

In the general population, exercise can increase the immediate risk of SCD, even in habitual exercisers, yet habitual exercise lowers the lifetime risk of SCD and mortality compared to those who do not exercise regularly--termed the “paradox of exercise”. ^37^ This is consistent with the role of the autonomic nervous system in arrhythmogenesis. Catecholamines are arrhythmogenic, while vagal tone, which increases with regular exercise, is protective against ventricular arrhythmia. ^38^ In the current study, 3 individuals had SCA during vigorous exercise. However, SCA and SCD occurred predominantly at other times, regardless of engagement in vigorous exercise. One national-claims-database analysis suggested an association of vigorous exercise with lower mortality in an older population of individuals with HCM, although detailed exercise and clinical data were not available.^39^ The current study did not show graded improvement in mortality with increasing exercise as shown in the general population. However, there was no increase in mortality or total arrhythmic endpoints seen, and the relatively young age and short timeframe may not have allowed demonstration of the longer-term benefits of exercise.

Whether there is an “optimal” duration, frequency, and/or intensity of exercise for individuals with HCM cannot be determined from these data, as the purpose of the study was to provide information on risk for those wishing to exercise, and the study was not powered to show superiority in any exercise classification group. In the general population, most studies show a graded benefit with increasing intensity of exercise, ^40, 41^ although some show a “J shaped curve” with higher mortality at highest levels of intensity. ^42^ More than one third of participants were instructed by physicians to perform no exercise at all, or only light-intensity activities. These data do not support this overly conservative approach. However, future studies will be needed to determine what exercise volume and intensity will be most beneficial for patients with HCM.

The rationale for including the genotype-positive/phenotype-negative group in the prespecified primary analysis group was lingering uncertainty about risk in these individuals, which our data have shown, significantly impacts patient care. Close to half of genotype positive, phenotype negative participants reported receiving recommendations for some or complete exercise restriction. Although engaging in vigorous exercise and competitive sports is considered reasonable for genotype-positive/phenotype-negative individuals by US and European guidelines (Class 2a), both documents acknowledge limited data in this group. ^7^ Recent molecular autopsy series suggest a potentially non-zero risk of SCD for these individuals, with identification of pathogenic genetic variants for HCM in 5-10% of young SCD victims despite absence of hypertrophy or structural abnormalities. ^11, 12^ As it is unclear whether these genetic findings were the primary trigger for SCD, prospective outcome studies in genotype positive/phenotype negative individuals continue to be warranted. The absence of events in this group in the present study is reassuring and supportive of current guideline recommendations, but continued surveillance of these individuals is advisable.

As fewer patients exercising vigorously were symptomatic compared to those who exercise moderately or were sedentary, post-hoc analysis was performed in asymptomatic patients with overt HCM (71%), excluding those with exertional symptoms which may have precluded more intense exercise. Even after removing symptomatic patients, the point-estimate for vigorous exercise was similar, and neither exercise group was superior to the other. While we cannot exclude the possibility that patients exercising vigorously were less severely affected, it is also possible that deconditioning due to exercise restriction may have increased symptomatic burden, as suggested by improvements in physical functioning and quality of life seen in the RESET trial.

### Limitations

LIVE participants may not be representative of all patients with HCM. While recruitment materials were aimed at individuals engaging in any level of physical activity, the percentage of those engaged in vigorous exercise was higher than expected based on prior surveys of patients with HCM^5^ as well as data on exercise practices in Americans in general. ^23 43^ It is likely that individuals interested in exercise were more interested in study participation. However, the over-representation of individuals engaged in vigorous exercise allowed robust comparisons and exploratory subgroup analyses.

While almost a quarter of participants were self-enrolled, most had received care at high-volume HCM centers with expertise in management and risk-assessment of patients with HCM, as recommended by current guidelines.^7^ Whether the data can be extrapolated to patients managed outside of these centers cannot be determined.

The percentage of participants with ICDs is higher than in most published HCM cohorts. ^2^ While the reason for this is unclear, prior data on safety of sports for individuals with HCM and ICDs may have led to higher enrollment of participants with ICDs who were more active than the average patients with HCM.

Individuals exercising vigorously differed in some ways from those exercising less vigorously or were sedentary. However, most known risk factors for sudden death did not differ between the groups, and controlling for those that did, did not impact the findings. The possibility of survival bias, (which could go in either direction,) cannot be excluded. However, while the subgroup of younger individuals (14-22 years) was too small for meaningful analysis, it is reassuring that there were fewer events in the competitive/other vigorous exercisers than those exercising moderately or sedentary.

### Conclusion

Among individuals with HCM, or those who are genotype-positive/phenotype - negative, who are managed in experienced centers, those exercising vigorously did not experience a higher rate of death or life-threatening arrhythmias than those exercising moderately or the sedentary. These data will inform discussion between patients and physicians regarding vigorous exercise participation, in the context of overall expert assessment and management of HCM, using an individualized shared decision-making framework.

## Supplementary Material

Supplemental figures and tables

## Figures and Tables

**Table 1:** Baseline demographic, genetic, and clinical data

	Non-Vigorous	Vigorous	Cohen's d or h	Vigorous non-competitive	Vigorous competitive	Cohen's d or h
**N**	(N = 961)	(N = 699)		(N = 440)	(N = 259)	
**Age, mean (SD) years**	40.5 (13.9)	36.1 (15.3)	0.31	40.0 (13.5)	29.3 (15.8)	0.74
**Age**						
< 18	101 (10.5%)	121 (17.3%)	-0.20	28 (6.4%)	93 (35.9%)	-0.77
18-25	67 (7.0%)	82 (11.7%)	-0.16	51 (11.6%)	31 (12.0%)	-0.01
> 25	793 (82.5%)	496 (71.0%)	0.28	361 (82.0%)	135 (52.1%)	0.65
**Sex**						
Male	518 (53.9%)	467 (66.8%)	-0.26	298 (67.7%)	169 (65.3%)	0.05
Female	443 (46.1%)	232 (33.2%)	0.26	142 (32.3%)	90 (34.7%)	-0.05
**Race**						
White	856 (89.1%)	631 (90.3%)	-0.04	393 (89.3%)	238 (91.9%)	-0.09
Black	36 (3.7%)	22 (3.1%)	0.03	13 (3.0%)	9 (3.5%)	-0.03
Other	69 (7.2%)	46 (6.6%)	0.02	34 (7.7%)	12 (4.6%)	0.13
Hispanic/Latino	49 (5.1%)	32 (4.6%)	0.02	17 (3.9%)	15 (5.8%)	-0.09
**Genotype**						
Positive	415 (43.2%)	330 (47.2%)	-0.08	196 (44.5%)	134 (51.7%)	-0.14
Variant Uncertain	74 (7.7%)	64 (9.2%)	-0.05	39 (8.9%)	25 (9.7%)	-0.03
Significance						
Negative	153 (15.9%)	120 (17.2%)	-0.03	80 (18.2%)	40 (15.4%)	0.07
Unknown/not tested	319 (33.2%)	185 (26.5%)	0.15	125 (28.4%)	60 (23.2%)	0.12
**Family History**						
FH SCD/resuscitated arrest	378 (39.3%)	281 (40.2%)	-0.02	182 (41.4%)	99 (38.2%)	0.07
FH HCM	537 (55.9%)	401 (57.4%)	-0.03	250 (56.8%)	151 (58.3%)	-0.03
**Phenotype negative**	52 (5.4%)	74 (10.6%)	-0.19	26 (5.9%)	48 (18.5%)	-0.40
						
**Overt HCM (N, %)**	**909 (94.6%)**	**625 (89.4%)**	**0.19**	**414 (94.5%)**	**211 (71.9%)**	**0.40**
**Age at Diagnosis, mean, SD, years**	31.8 (15.6)	30.1 (15.8)	0.11	32.1 (15.3)	26.2 (16.2)	-0.38
**Mode of Diagnosis**						
Symptoms	376 (41.4%)	233 (37.3%)	0.08	160 (38.6%)	73 (34.6%)	0.08
Family screening	223 (24.5%)	165 (26.4%)	-0.04	110 (26.6%)	55 (26.1%)	0.01
ECG screening	31 (3.4%)	31 (5.0%)	-0.08	25 (6.0%)	6 (2.8%)	0.16
Incidental/other	317 (34.9%)	223 (35.7%)	-0.02	139 (33.6%)	84 (39.8%)	-0.13
**Apical morphology**	79 (8.7%)	105 (16.8%)	-0.25	63 (15.2%)	42 (19.9%)	-0.12
**History of cardiac arrest**	31 (3.4%)	36 (5.8%)	-0.12	20 (4.8%)	16 (7.6%)	-0.12
**Exertional symptoms**	271 (29.8%)	90 (14.4%)	0.38	64 (15.5%)	26 (12.3%)	0.09
**History of Syncope**	231 (25.4%)	141 (22.6%)	0.07	106 (25.6%)	35 (16.6%)	0.22
**History of NSVT^a^**	247 (27.2%)	147 (23.5%)	0.09	109 (26.3%)	38 (18.0%)	0.20
**Myectomy**	200 (22.0%)	88 (14.1%)	0.21	70 (16.9%)	18 (8.5%)	0.26
**ICD^b^**	397 (43.7%)	254 (40.6%)	0.06	185 (44.7%)	69 (32.7%)	0.25
**Pacemaker**	12 (1.3%)	4 (0.6%)	0.07	4 (1.0%)	0	0.20
**Secondary prevention ICD indication (% of those with ICD)**	47 (5.2%)	45 (7.2%)	0.08	29 (15.7%)	16 (23.1%)	-0.19
**LV^c^ maximal wall thickness, mean, (SD), mm**	21.5 (12.2)	20.0 (6.3)	0.15	19.9 (6.2)	20.2 (6.6)	-0.05
LVEF^d^,%, mean**	66.2 (7.7)	66.1 (7.1)	0.01	65.9 (7.3)	66.7 (6.7)	-0.11
**LVOT^e^ rest gradients, mean, mmHg**	23.0 (25.9)	18.3 (22.3)	0.19	19.2 (23.4)	16.5 (19.9)	0.12
**≥ 30 mmHg, N, (%)**	164 (22.6%)	68 (14.1%)	0.22	50 (15.4%)	18 (11.5%)	0.11
**LVOT provoked gradients (Valsalva or exercise), mean, mmHg**	51.1 (46.3)	39.6 (42.0)	0.26	39.8 (41.2)	39.2 (43.9)	0.02
**≥ 30 mmHg**	279 (54.2%)	135 (42.2%)	0.24	94 (42.7%)	41 (41.0%)	0.03
**Late gadolinium enhancement**						
None	691 (76.0%)	472 (75.5%)	0.01	312 (75.4%)	160 (75.8%)	-0.01
Mild/moderate/patchy/<15%	161 (17.7%)	110 (17.6%)	0.00	69 (16.7%)	41 (19.4%)	-0.07
Extensive/>15%	57 (6.3%)	43 (6.9%)	-0.02	33 (8.0%)	10 (4.7%)	0.14

Gray shaded variables are presented for participants with overt HCM only All values presented as N (%) unless otherwise indicated

a NSVT, nonnsustained ventricular tachycardia,

b ICD, implantable cardioverter defibrillator,

c LV, left ventricular,

d EF, ejection fraction,

e OT, outflow tract

**Table 2:** Endpoint events

	Non-Vigorous N=961	Vigorous N=699	Total N=1660	Vigorous Competitive N=259	Vigorous Non-competitive N=440
**TOTAL composite endpoint**					
N	44	33	77	10	23
Rate per 1,000 person-year (95% CI)	15.3 (11.4, 20.5)	15.9 (11.3, 22.4)	15.6 (12.5, 19.6)	13.1 (7.0, 24.0)	17.6 (11.7, 26.5)
**Individual Endpoints**					
Death*					
N	8	4	12	1	3
Rate per 1,000 person-year (95% CI)	2.7 (1.4, 5.5)	1.9 (0.7, 5)	2.3 (1.2, 4)	1.3 (0.2, 9)	2.2 (0.7, 7.0)
**Cardiac Arrest**					
N	2	4	6	3	1
Rate per 1,000 person-year (95% CI)	0.7 (0.2, 2.7)	1.9 (0.7, 5.0)	1.1 (0.5, 2.6)	3.9 (1.2, 12)	0.7 (0.1, 5.0)
**Arrhythmic Syncope, patients with ICD**					
N	19	15	34	2	13
Rate per 1,000 person-year (95% CI)	6.6 (4.2, 10.3)	7.1 (4.3, 11.8)	6.8 (4.9, 9.6)	2.6 (0.6, 10.3)	9.8 (5.7, 16.9)
**Appropriate ICD Shock (no syncope)**					
N	11	8	19	1	7
Rate per 1,000 person-year (95% CI)	3.8 (1.9, 7.6)	3.8 (1.9, 7.6)	3.8 (2.4, 6.0)	1.3 (0.2, 9.1)	5.3 (2.5, 11.0)
**Definite or likely arrhythmic Syncope, patients without ICD**					
N	8	7	15	3	4
Rate per 1,000 person-year (95% CI)	2.7 (1.4, 5.5)	3.3 (1.6, 6.9)	3.0 (1.8, 5.0)	3.9 (1.2, 12.0)	3.0 (1.1, 8.0)

* Includes 8 SCD and 4 noncardiac deaths

**Table 3:** Characteristics of individuals suffering sudden cardiac death or cardiac arrest

Exercise group	Event	Enrollment Age	Sex	ICD^a^	Risk factors	Activity with event
Sedentary	SCA^b^	12.6	M	yes		standing in line at school
Sedentary	SCD^c^	15.5	M			sleep
Sedentary	SCD	53.5	M		syncope	sleep
						
Moderate	SCA	8.9	M			jogging/warmup for karate
Moderate	SCD	21.9	M		syncope, NSVT^c^	sleep
Moderate	SCD	31.6	M	yes		hunting
Moderate	SCD	35.7	M		FH^d^ SCD, NSVT	unknown
						
Vigorous	SCA	9.5	M			sitting at assembly
Vigorous competitive	SCA	18.4	M			running recreationally
Vigorous	SCD	23.9	M		FH SCD	home alone
Vigorous competitive	SCA	35.4	M			cycling recreationally
Vigorous	SCD	53.2	M			unknown
Vigorous competitive	SCA	54.6	M			sand volleyball, between games
Vigorous competitive	SCD	57.1	M	Yes		driving car

a Number of appropriate ICD therapies not associated with cardiac arrest appear in Table 2

b SCA, (resuscitated) cardiac arrest, ^a^SCD, sudden cardiac death, ^c^NSVT, nonsustained ventricular tachycardia, ^d^FH, family history

## Key Points

Question: Does vigorous exercise increase risk of mortality or ventricular arrhythmia in individuals with hypertrophic cardiomyopathy (HCM)?

Findings: Individuals exercising vigorously did not have higher mortality or a higher incidence of ventricular arrhythmias, with a hazard ratio of 1.01 in comparison with those not exercising vigorously, with the upper confidence level not exceeding the pre-specified boundary for inferiority.

Meaning: These data do not support universal restriction of vigorous exercise for individuals with HCM.
