# Supplementary material for: Vigorous Exercise in Patients with Hypertrophic Cardiomyopathy: Results of the Prospective, Observational, Multinational, “Lifestyle and Exercise in HCM” (LIVE-HCM) Study
Source: JAMA Cardiol. Author manuscript; Available in PMC 2023 Jun 15. (PMC10193262; doi:10.1001/jamacardio.2023.1042)
Supplement: Supplemental figures and tables [file EMS176388-supplement-Supplemental_figures_and_tables.docx]

**Supplement table of Contents (in order of appearance in manuscript)**

P 2 Supplemental Figure 1. Consort diagram

P 3-4 Supplemental Table 1. Baseline Demographic and Clinical data for Sedentary, Moderate, and Vigorous Groups

P 5-6 Supplemental Table 2. Sports Participation in Vigorous-Competitive Subgroup

P 7 Supplemental Table 3. Endpoint events In Sedentary, Moderate, and Vigorous Groups

P 8 Supplemental Figure 2. Survival free of death, cardiac arrest, appropriate ICD shock, or arrhythmic syncope in Sedentary, Moderate, and Vigorous Groups

P 9-11 Supplemental Table 4. Baseline Demographic and Clinical Data for Participants Age 14-22 years (Varsity/Traveling, Other Vigorous, and

Moderate/Sedentary)

P 12 Supplemental Table 5. Endpoint Events in Participants Age 14-22 years (Varsity/Traveling, Other Vigorous, and Moderate/Sedentary)

P 13-14 List of sites and site principal investigators

**Supplemental Figure 1. CONSORT diagram**

**
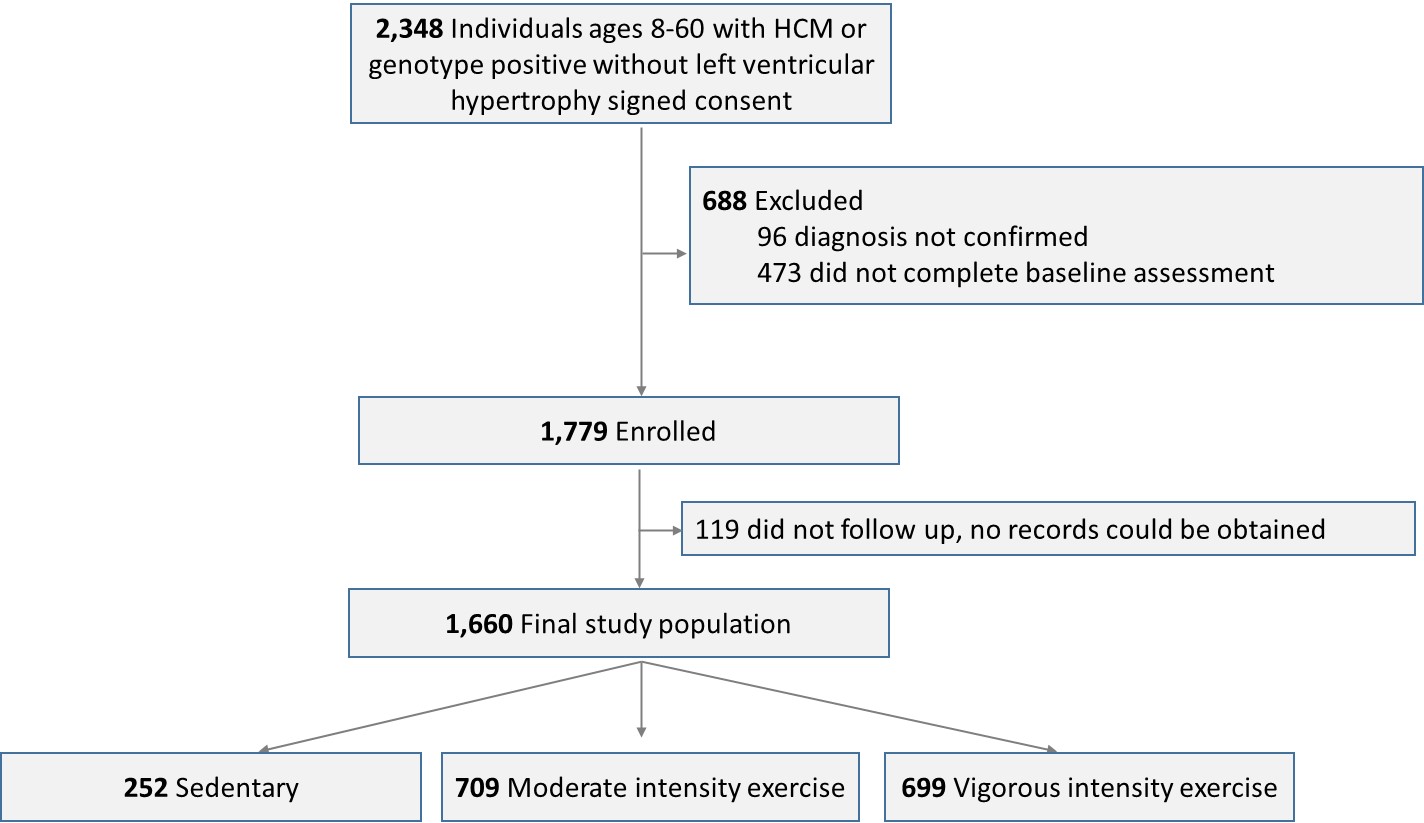
**

**Supplemental Table 1. Baseline Demographic and Clinical data for Sedentary, Moderate, and Vigorous Groups**

|  |  |  |  |  |  |
| --- | --- | --- | --- | --- | --- |
|  | **Sedentary^1^** | **Moderate^2^** | **Vigorous^3^** | **Cohen’s d or h**  **1 vs 2** | **Cohen’s d or h**  **1 vs 3** |
| **N** | (N = 252) | (N = 709) | (N = 699) |  |  |
| **Age, mean (SD)** | 41.5 (13.8) | 40.2 (13.9) | 36.1 (15.3) | -0.10 | 0.37 |
| **Age** |  |  |  |  |  |
| < 18 | 26 (1.3%) | 75 (1.6%) | 121 (17.3%) | -0.03 | -0.63 |
| 18-25 | 14 (5.6%) | 53 (7.5%) | 82 (11.7%) | -0.08 | -0.22 |
| > 25 | 212 (84.1%) | 581 (81.9%) | 496 (71.%) | 0.06 | 0.32 |
| **Sex** |  |  |  |  |  |
| Male | 118 (46.8%) | 400 (56.4%) | 467 (66.8%) | -0.19 | -0.41 |
| Female | 134 (53.2%) | 309 (43.6%) | 232 (33.2%) | 0.19 | 0.41 |
| **Race** |  |  |  |  |  |
| White | 217 (86.1%) | 639 (90.1%) | 631 (90.3%) | -0.12 | -0.13 |
| Black | 15 (6.0%) | 21 (3.0%) | 22 (3.1%) | 0.15 | 0.14 |
| Other | 20 (7.9%) | 49 (6.9%) | 46 (6.6%) | 0.04 | 0.05 |
| Hispanic/Latino | 14 (5.6%) | 35 (4.9%) | 32 (4.6%) | 0.03 | 0.05 |
| **Genotype** |  |  |  |  |  |
| Positive | 112 (44.4%) | 33 (42.7%) | 330 (47.2%) | 0.03 | -0.06 |
| Variant of uncertain significance | 16 (6.3%) | 58 (8.2%) | 64 (9.2%) | -0.07 | -0.11 |
| Negative | 41 (16.3%) | 112 (15.8%) | 120 (17.2%) | 0.01 | -0.02 |
| Unknown/not tested (All others) | 83 (32.9%) | 236 (33.3%) | 185 (26.5%) | -0.01 | 0.14 |
| **Family History** |  |  |  |  |  |
| FH SCD/resuscitated arrest | 94 (37.3%) | 284 (4.1%) | 281 (40.2%) | 0.91 | -0.06 |
| FH HCM | 138 (54.8%) | 399 (56.3%) | 401 (57.4%) | -0.03 | -0.05 |
|  |  |  |  |  |  |
| **Phenotype Negative (N, %)** | 5 (2.0%) | 47 (6.6%) | 74 (1.6%) | -0.24 | 0.03 |
|  |  |  |  |  |  |
| **Overt HCM (N, %)** | **247 (98.0%)** | **662(93.4%)** | **625 (89.4%)** | 0.24 | 0.38 |
| **Age at Diagnosis** | 32.1 (16.2) | 31.7 (15.3) | 30.1 (15.8) | 0.03 | 0.12 |
| **Mode of Diagnosis** |  |  |  |  |  |
| Symptoms | 106 (42.9%) | 270 (40.8%) | 233 (37.3%) | 0.04 | 0.11 |
| Family screening | 54 (21.9%) | 169 (25.5%) | 165 (26.4%) | -0.08 | -0.11 |
| ECG screening | 10 (4.0%) | 21 (3.2%) | 31 (5.0%) | 0.04 | -0.05 |
| Other (all others) | 85 (34.4%) | 232 (35.0%) | 223 (35.7%) | -0.01 | -0.03 |
| **Apical morpholoyg** | 20 (8.1%) | 59 (8.9%) | 105 (16.8%) | -0.03 | -0.27 |
| **Hx cardiac arrest** | 9 (3.6%) | 22 (3.3%) | 36 (5.8%) | 0.02 | -0.10 |
| **Exertional dyspnea and/or CP** | 80 (32.3%) | 191(28.9%) | 90 (14.4%) | 0.07 | 0.43 |
| **History of Syncope** | 64 (25.9%) | 167 (25.2%) | 141 (22.6%) | 0.02 | 0.08 |
| **History of NSVT^a^** | 61 (24.7%) | 186 (28.1%) | 147 (23.5%) | -0.08 | 0.03 |
| **Myectomy** | 65 (26.3%) | 135 (20.4%) | 88 (14.1%) | 0.14 | 0.31 |
| **ICD^b^** | 123 (49.8%) | 274 (41.4%) | 254 (40.6%) | 0.17 | 0.19 |
| **Pacemaker** | 3 (1.2%) | 9 (1.4%) | 4 (0.6%) | -0.02 | 0.06 |
| **Secondary Prevention CD indication (% of those with ICD)** | 16 (13%) | 31 (11.3%) | 45 (17.7%) | 0.05 | -0.13 |
| **LV^c^ Maximal wall thickness (mm)** | 21.9 (14.4) | 21.4 (11.4) | 20.0 (6.3) | 0.04 | 0.20 |
| **LV EF mean**** | 65.2 (8.6) | 66.6 (7.3) | 66.1 (7.1) | -0.19 | -0.13 |
| **LVOT^d^ rest gradients, mmHg** | 24.7(28.1) | 22.4(25.1) | 18.3(22.3) | 0.09 | 0.26 |
| ≥ 35 mmHg | 42(21.8%) | 100(18.8%) | 57(11.9%) | 0.07 | 0.27 |
| **LVOT gradient (Valsalva or exercise), mean, mmHg** | 50.2(45.8) | 51.4(46.6) | 39.6(42.0) | -0.03 | 0.25 |
| ≥ 35 mmHg | 74(51.4%) | 191(51.5%) | 121(37.8%) | 0.00 | 0.27 |
| **LGE****** |  |  |  |  |  |
| None | 191 (77.3%) | 500 (75.5%) | 472 (75.5%) | 0.04 | 0.04 |
| Mild/mod/patchy/<15% | 40 (16.2%) | 121 (18.3%) | 110 (17.6%) | -0.06 | -0.04 |
| Extensive/>15% | 16 (6.5%) | 41 (6.2%) | 43 (6.9%) | 0.01 | -0.02 |

Gray shaded variables are presented for participants with overt HCM only; All values presented as N (%) unless otherwise indicated

**^a^**NSVT, nonnsustained ventricular tachycardia, **^b^**ICD, implantable cardioverter defibrillator, **^c^**LV, left ventricular, **^d^**EF, ejection fraction, **^e^**OT, outflow tract

**Supplemental Table 2. Sports participation in vigorous competitive group**

| **AGE (years)** | **8 to 13** | **14-22** |  | **23-60** |
| --- | --- | --- | --- | --- |
|  |  | **varsity/JV/traveling** | **league/**  **organized competition** |  |
| Baseball/Softball | 14 | 13 | 1 | 17 |
| Basketball | 9 | 13 | 8 | 13 |
| Bicycling | 0 | 0 | 2 | 16 |
| cheerleading | 2 | 1 | 0 | 0 |
| cricket | 0 | 0 | 0 | 2 |
| crossfit | 0 | 0 | 0 | 1 |
| dance (comp) | 2 | 0 | 0 | 0 |
| discus | 0 | 1 | 0 | 0 |
| diving | 0 | 1 | 0 | 0 |
| equestrian | 2 | 0 | 2 | 1 |
| football-tackle | 2 | 3 | 0 | 0 |
| football touch/flag | 5 | 0 | 2 | 3 |
| frisbee | 0 | 0 | 2 | 4 |
| gymnastics | 2 | 0 | 1 | 0 |
| ice hockey | 1 | 2 | 1 | 10 |
| lacrosse | 3 | 1 | 0 | 0 |
| martial arts | 5 | 0 | 0 | 0 |
| "tuff mudder | 0 | 0 | 0 | 4 |
| raquetball | 0 | 0 | 0 | 2 |
| rowing | 0 | 2 | 0 | 0 |
| running/track | 3 | 11 | 4 | 33 |
| sailing | 0 | 0 | 1 | 4 |
| ski racing | 2 | 0 | 1 | 0 |
| soccer | 10 | 5 | 2 | 16 |
| swimming | 4 | 3 | 1 | 10 |
| tennis | 1 | 4 | 0 | 6 |
| volleyball | 4 | 5 | 3 | 11 |
| race walking | 0 | 0 | 0 | 7 |
| other | 1 | 0 | 2 | 8 |

Total numbers of sports are greater than individuals as some participated in more than one sport

**Supplemental Table 3. Endpoint events In Sedentary, Moderate, and Vigorous Groups**

|  | Sedentary | Moderate | Vigorous |  |
| --- | --- | --- | --- | --- |
|  | N=252 | N=709 | N=699 |  |
| TOTAL composite endpoint |  |  |  |  |
| N | 15 | 29 | 33 |  |
| Rate per 1,000 person-year  (95% CI) | 20.2  (12.2, 33.4) | 13.6  (9.4, 19.6) | 15.9  (11.3, 22.4) |  |
|  |  |  |  |  |
| Individual Endpoints |  |  |  |  |
| Death* |  |  |  |  |
| N | 3 | 5 | 4 |  |
| Rate per 1,000 person-year  (95% CI) | 3.9  (1.3, 12.2) | 2.3  (1.0, 5.5) | 1.9  (0.7, 5) |  |
| Cardiac Arrest |  |  |  |  |
| N | 1 | 1 | 4 |  |
| Rate per 1,000 person-year  (95% CI) | 1.3  (0.2, 9.0) | 0.5  (0.1, 3.3) | 1.9  (0.7, 5.0) |  |
| Arrhythmic Syncope, patients with ICD |  |  |  |  |
| N | 6 | 13 | 15 |  |
| Rate per 1,000 person-year  (95% CI) | 8.0  (3.6, 17.8) | 6.0  (3.5, 10.4) | 7.1  (4.3, 11.8) |  |
| Appropriate ICD Shock (no syncope) |  |  |  |  |
| N | 3 | 3 | 8 |  |
| Rate per 1,000 person-year  (95% CI) | 3.9  (1.3, 12.2) | 3.7  (1.9, 7.4) | 3.8  (1.9, 7.6) |  |
| Definite or likely arrhythmic Syncope, patients without ICD |  |  |  |  |
| N | 3 | 5 | 7 |  |
| Rate per 1,000 person-year  (95% CI) | 3.9  (1.3, 12.2) | 2.3  (1.0, 5.5) | 3.3  (1.6, 6.9) |  |

* Includes 8 SCD and 4 noncardiac deaths

**Supplemental Figure 2. Kaplan-Meier survival curve for freedom from composite endpoint** **(death, cardiac arrest, appropriate ICD shock, or arrhythmic syncope) by exercise group**


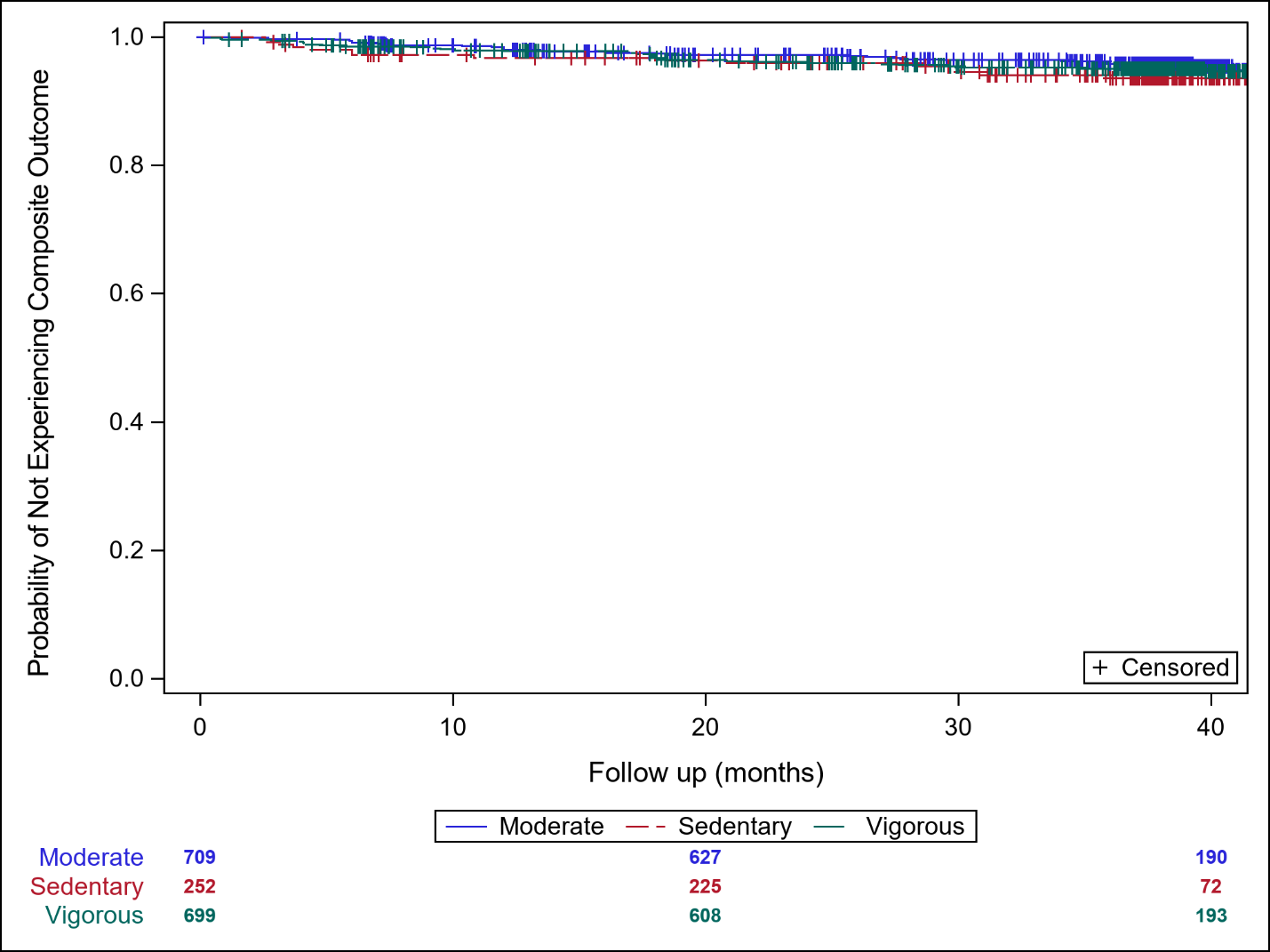


**Supplemental Table 4: Baseline Demographic and Clinical Data for Participants Age 14-22 years**

|  | **Non-Vigorous ^1^** | **Other Vigorous ^2^** | **V/JV/Travel ^3^** | **Cohen’s d or h**  **1 vs 2** | **Cohen’s d or h**  **1 vs 3** |
| --- | --- | --- | --- | --- | --- |
| **N** | (N = 97) | (N = 50) | (N = 56) |  |  |
| **Age, mean (SD) years** | 17.7 (2.3) | 18.6 (2.3) | 17.0 (1.6) | -0.38 | 0.33 |
| **Age** |  |  |  |  |  |
| < 18 | 55 (56.7%) | 19 (38.0%) | 41 (73.2%) | 0.38 | -0.35 |
| 18-25 | 42 (43.3%) | 31 (62.0%) | 15 (26.8%) | -0.38 | 0.35 |
| **Sex** |  |  |  |  |  |
| Male | 53 (54.6%) | 35 (70.0%) | 32 (57.1%) | -0.32 | -0.05 |
| Female | 44 (45.4%) | 15 (30.0%) | 24 (42.9%) | 0.32 | 0.05 |
| **Race** |  |  |  |  |  |
| White | 82 (84.5%) | 42 (84.0%) | 44 (78.6%) | 0.01 | 0.15 |
| Black | 5 (5.2%) | 3 (6.0%) | 5 (8.9%) | -0.03 | -0.15 |
| Other | 10 (10.3%) | 5 (10.0%) | 7 (12.5%) | 0.01 | -0.07 |
| Hispanic/Latino | 9 (9.3%) | 7 (14.0%) | 2 (3.6%) | -0.15 | 0.24 |
| **Genotype** |  |  |  |  |  |
| Positive | 51 (52.6%) | 26 (52.0%) | 36 (64.3%) | 0.01 | -0.24 |
| Variant Uncertain Significance | 5 (5.2%) | 6 (12.0%) | 5 (8.9%) | -0.25 | -0.15 |
| Negative | 17 (17.5%) | 8 (16.0%) | 8 (14.3%) | 0.04 | 0.09 |
| Unknown/not tested | 24 (24.7%) | 10 (20.0%) | 7 (12.5%) | 0.11 | 0.32 |
| **Family History** |  |  |  |  |  |
| FH SCD/resuscitated arrest | 34 (35.1%) | 22 (44.0%) | 17 (30.4%) | -0.18 | 0.10 |
| FH HCM | 58 (59.8%) | 31 (62.0%) | 39 (69.6%) | -0.05 | -0.21 |
|  |  |  |  |  |  |
| **Phenotype negative** | 6 (6.2%) | 5 (10.0%) | 14 (25.0%) | -0.14 | -0.54 |
|  |  |  |  |  |  |
| **Overt HCM (N, %)** | **91 (93.8%)** | **45 (90.0%)** | **42 (75.0%)** | 0.14 | 0.54 |
| **Age at Diagnosis, mean, SD, years** | 10.3 (5.5) | 13.0 (6.2) | 14.2 (4.3) | -0.32 | -0.76 |
| **Mode of Diagnosis** |  |  |  |  |  |
| Symptoms | 18 (19.8%) | 29 (33.3%) | 20 (37.0%) | -0.31 | -0.39 |
| Family screening | 27 (29.7%) | 27 (31.0%) | 17 (31.5%) | -0.03 | -0.04 |
| ECG screening | 2 (2.2%) | 2 (2.3%) | 1 (1.9%) | -0.01 | 0.02 |
| Incidental/other | 45 (49.5%) | 32 (36.8%) | 18 (33.3%) | 0.26 | 0.33 |
| **Apical morphology** | 4 (4.4%) | 9 (10.3%) | 7 (13.0%) | -0.23 | -0.32 |
| **History of cardiac arrest** | 8 (8.8%) | 7 (8.0%) | 6 (11.1%) | 0.03 | -0.08 |
| **Exertional symptoms** | 12 (13.2%) | 8 (9.2%) | 4 (7.4%) | 0.13 | 0.19 |
| **History of Syncope** | 22 (24.2%) | 17 (19.5%) | 8 (14.8%) | 0.11 | 0.24 |
| **History of NSVT^a^** | 16 (17.6%) | 11 (12.7%) | 6 (11.1%) | 0.14 | 0.19 |
| **Myectomy** | 19 (20.9%) | 8 (9.2%) | 3 (5.6%) | 0.33 | 0.47 |
| **ICD^b^** | 42 (46.2%) | 40 (46.0%) | 19 (35.2%) | 0.00 | 0.22 |
| **Secondary prevention ICD indication (% of those with ICD)** | 7 (7.7%) | 3(6.7%) | 5(11.9%) | 0.04 | -0.14 |
| **LV^c^ maximal wall thickness, mean, (SD), mm** | 23.4 (10.8) | 23.7 (9.3) | 20.9 (7.1) | -0.03 | 0.25 |
| **LVEF^d^,%, mean**** | 67.9 (9.0) | 68.0 (7.3) | 67.9 (6.8) | -0.01 | 0.00 |
| **LVOT^e^ rest gradients, mean, mmHg** | 20.1 (24.0) | 17.6 (19.5) | 11.2 (4.4) | 0.11 | 0.43 |
| **≥ 30 mmHg, N, (%)** | 13 (19.1%) | 6 (17.6%) | 0 (0.0%) | 0.04 | 0.90 |
| **LVOT provoked gradients (Valsalva or exercise), mean, mmHg** | 39.4 (46.2) | 42.0 (55.0) | 28.6 (25.8) | -0.05 | 0.26 |
| **≥ 30 mmHg** | 9 (33.3%) | 5 (38.5%) | 5 (38.5%) | -0.11 | -0.11 |
| **Late gadolinium enhancement** |  |  |  |  |  |
| None | 73 (80.2%) | 37 (82.2%) | 29 (69.0%) | -0.05 | 0.26 |
| Mild/moderate/patchy/<15% | 13 (14.3%) | 7 (15.6%) | 12 (28.6%) | -0.04 | -0.35 |
| Extensive/>15% | 5 (5.5%) | 1 (2.2%) | 1 (2.4%) | 0.18 | 0.16 |

**Supplemental Table 5: Endpoint Events for Participants Age 14-22 years**

|  | Varsity/Travel | Other Vigorous | Mod + Sed | Total |  |
| --- | --- | --- | --- | --- | --- |
|  | N=56 | N=50 | N=97 | N=203 |  |

| **TOTAL composite endpoint** |  |  |  |  |
| --- | --- | --- | --- | --- |
| N  Rate per 1,000 person-year  (95% CI) | 1  5.7  (0.8, 40.8) | 0 | 6  20.7  (9, 46.2) | 7  11.4  (5.4, 24.0) |

| TOTAL composite endpoint |  |  |  |  |  |
| --- | --- | --- | --- | --- | --- |
| N | 1 | 0 | 6 | 7 |  |
|  |  |  |  |  |  |
| Individual Endpoints |  |  |  |  |  |
| Death* |  |  |  |  |  |
| N | 0 | 0 | 2 | 2 |  |
| Cardiac Arrest |  |  |  |  |  |
| N | 1 | 0 | 0 | 1 |  |
| Arrhythmic Syncope, patients with ICD |  |  |  |  |  |
| N | 0 | 0 | 3 | 3 |  |
| Appropriate ICD Shock (no syncope) |  |  |  |  |  |
| N | 0 | 0 | 0 | 0 |  |
| Definite or likely arrhythmic Syncope, patients without ICD |  |  |  |  |  |
| N | 0 | 0 | 1 | 1 |  |
|  |  |  |  |  |  |

* Includes 8 SCD and 4 noncardiac deaths

**List of Sites and Site Principal Investigators**

| **Site Name** | **Site Principal Investigator** |
| --- | --- |
| Baylor College of Medicine/Texas Children's Hospital | Silvana Molossi, MD, PhD |
| Boston Children's Hospital | Dominic Abrams, MD |
| Brigham and Women's Hospital | Carolyn Y Ho, MD |
| Children's Hospital of Philadelphia | Maully Shah, MBBS |
| Children's National Hospital | Charles I Berul, MD |
| Children's Omaha/University of Nebraska Medical Center | Christopher Erickson, MD |
| Cincinnati Children's Hospital | Richard Czosek,MD |
| Cleveland Clinic Heart, Vascular and Thoracic Institute | Peter Aziz, MD |
| Indiana University School of Medicine | Michael Emery, MD, Roopa Rao, MD |
| Johns Hopkins Hospital | Roselle Abraham, MD, Cindy James, PhD |
| Liverpool Heart and Chest Hospital | Robert M Cooper, MD |
| Lurie Children's Hospital | Gregory Webster, MD, MPH |
| Mayo Clinic | Michael Ackerman, MD, PhD |
| Nationwide Children's Hospital | Nandi Deipanjan, MD |
| New York University | Marina Cerrone, MD |
| Nicklaus Children's Hospital | Ronald Kantor, MD |
| Northwestern University Feinberg School of Medicine | Lubna Choudhury, MD |
| Oregon Health and Science University | Ahmad Masri, MD |
| Penn State Health/Milton S Hershey Medical Center | Eric Popjes, MD |
| Primary Children's Hospital | Susan Etheridge, MD |
| Royal Brompton Hospital/Imperial College, London | James Ware, MD |
| St. George's Hospital, NHS Foundation Trust | Maria Teresa Estaban Tome, MD, PhD |
| Stanford School of Medicine | Anne Dubin, MD |
| Starship Children's Hospital | Jonathan Skinner, MB ChB DCH MRCP |
| Toronto General Hospital | Michael J. Gollub, MD |
| Tufts Medical Center | Mark Link, MD |
| University of Arizona | Jill Tardif, MD |
| University of British Columbia Children's Hospital | Shubhayan Sanatani, MD |
| University of CA, Irvine/Childrens Hospital of Orange County | Anjan Batra, MD |
| University of California, San Francisco | Walter Li, MD |
| University of California,San Diego/ Rady's Children's Hospital | James Perry, MD |
| University of Iowa | Ian Law, MD |
| University of Louisville | Christopher Johnsrude, MD |
| University of Michigan | Sharlene Day, MD, Sara Saberi, MD, MS |
| University of Pittsburgh Medical Center | Timothy Wong, MD |
| University of Sydney/Cenetary Institute, Royal Prince Alfred Hospital | Chris Semsarian, MD |
| University of Tennessee | Jeffrey Tobin, MD |
| University of Texas, Southwestern | Aslan Turer, MD |
| University of Utah | Omar Wever-Pinzon, MD |
| Vanderbilt University | Prince Kannankeril, MD MSCI |
| Yale University | Rachel Lampert, MD |
